# Supplementary material for: Complexome profiling of the Chlamydomonas psb28 mutant reveals TEF5 as an early PSII assembly factor
Source: Plant Cell. 2025 Mar 17;37(6):koaf055. doi: 10.1093/plcell/koaf055 (PMC12164590; doi:10.1093/plcell/koaf055)
Supplement: koaf055_Supplementary_Data [file koaf055_supplementary_data.zip › psb28_interactive.zip!/plots/intensity/qualityPlots/peptides/_Cre08.g381702.t1.1.html]

### Cre08.g381702.t1.1

Trivial name:   
SeqCoverage [median]: 15.60  
Mass: 69.11 kDa  
  
 

| Sequence | MissedCleavage | Mass [Da] | wtIntensity | muIntensity | Score |
| --- | --- | --- | --- | --- | --- |
| AAIAAAHSVLPQWR | 0 | 1489.82 | 655.07 | 3006.08 | 84.36 |
| GIVASALR | 0 | 785.48 | 0.00 | 1118.11 | 91.91 |
| IGFTGSTAVGK | 0 | 1036.56 | 1312.04 | 1068.07 | 102.55 |
| KIGFTGSTAVGK | 1 | 1164.65 | 498.38 | 495.40 | 82.70 |
| LAEAVVGR | 0 | 813.47 | 307.51 | 963.48 | 101.64 |
| MLAAGAGAGVK | 0 | 944.51 | 0.00 | 1186.21 | 65.04 |
| TAGLIGGK | 0 | 715.42 | 0.00 | 1240.65 | 68.92 |
| TNLDEAAQATGLSKPAVSADLMSR | 1 | 2445.22 | 1813.23 | 4356.91 | 119.25 |
| VAGDVLEPPSR | 0 | 1138.60 | 769.58 | 609.15 | 142.43 |
|  |  |  | 5355.8 | 14044.1 |  |
